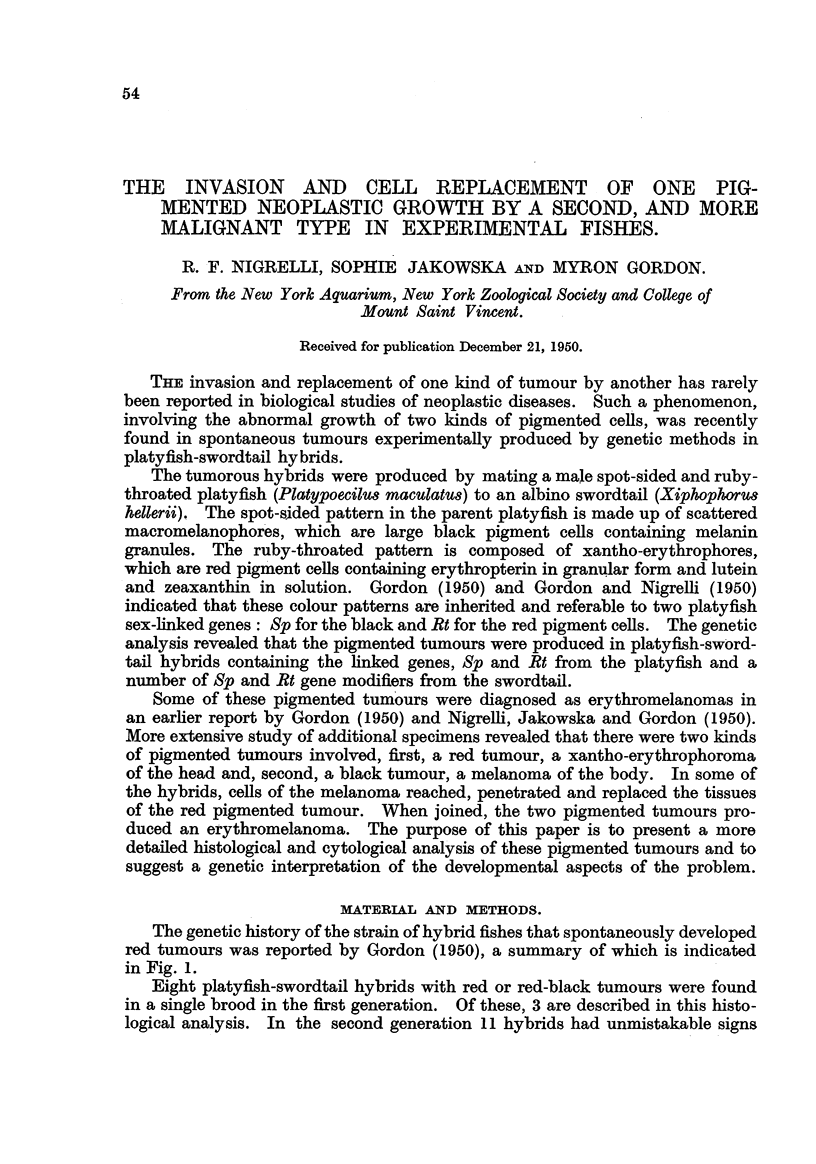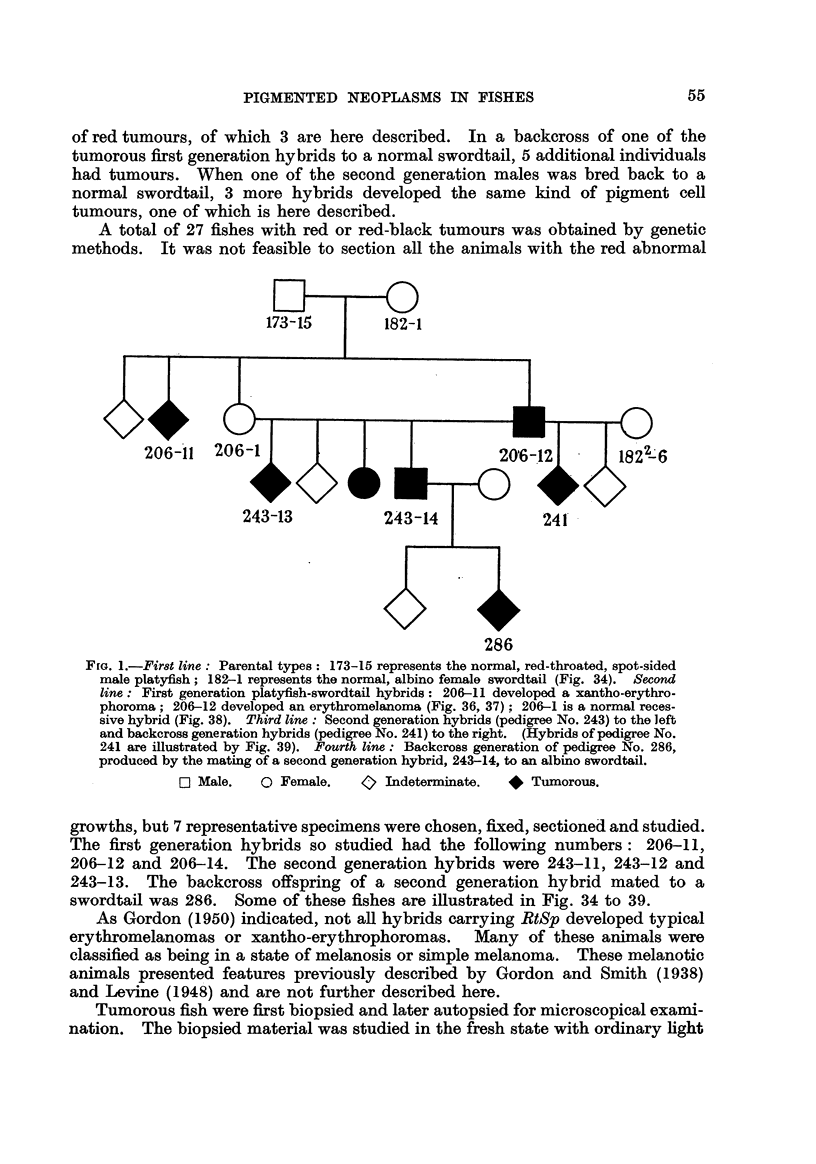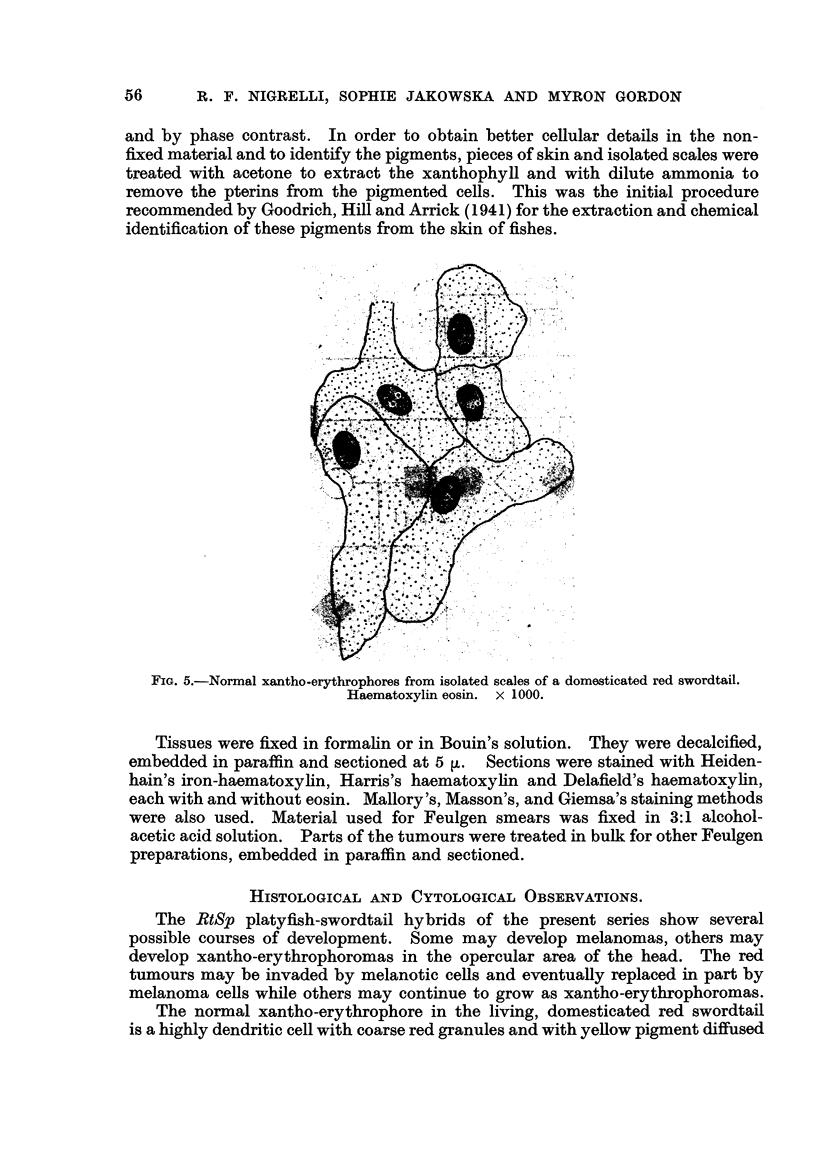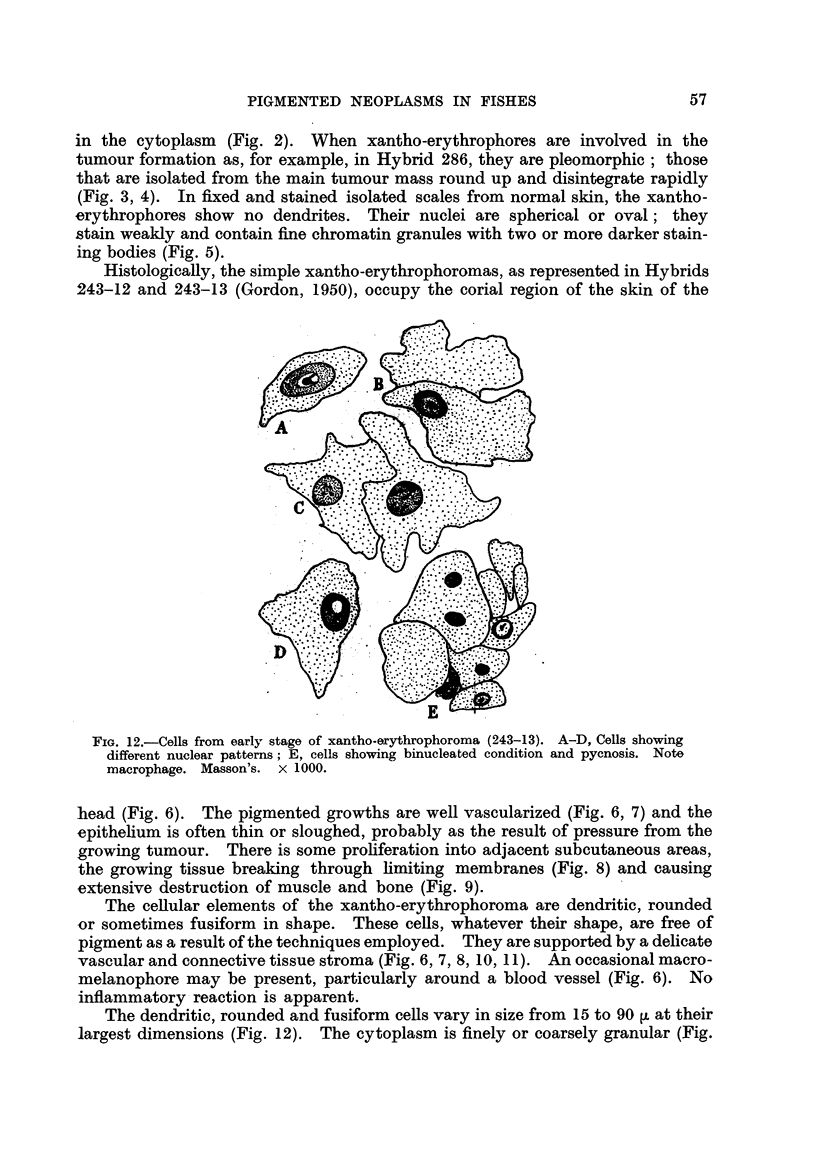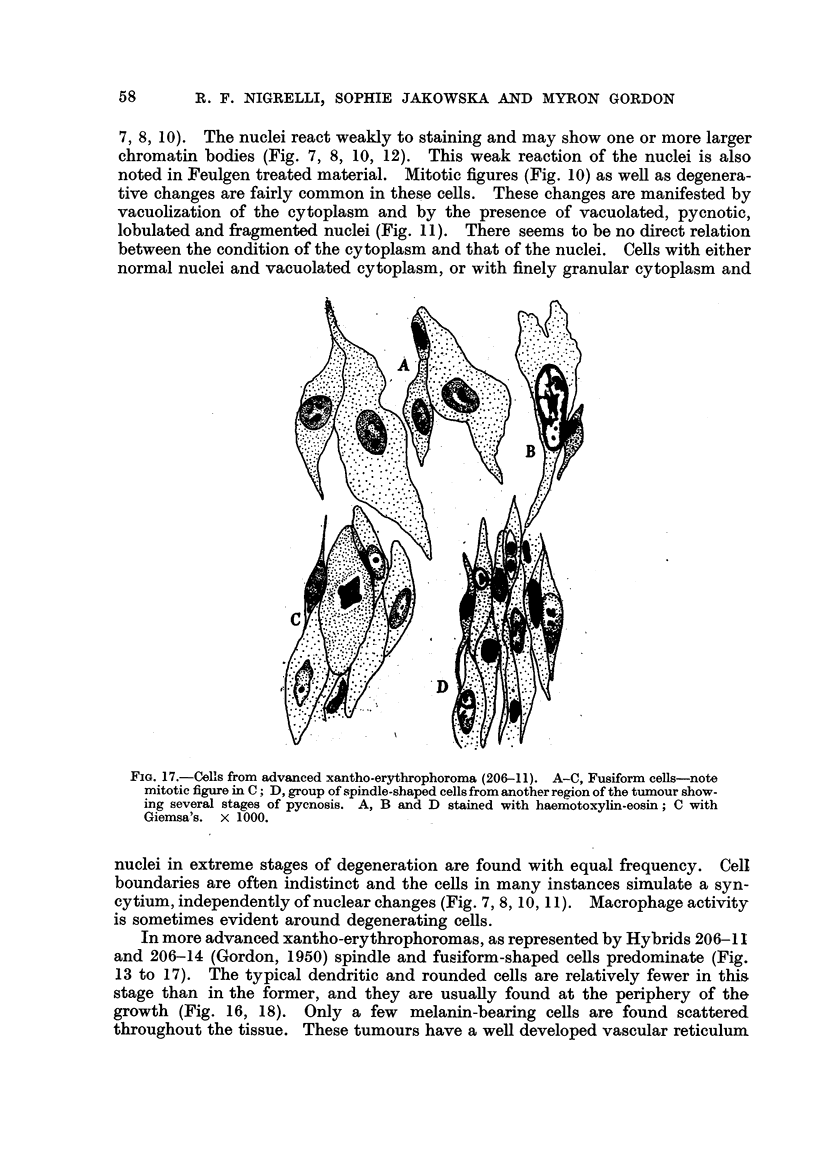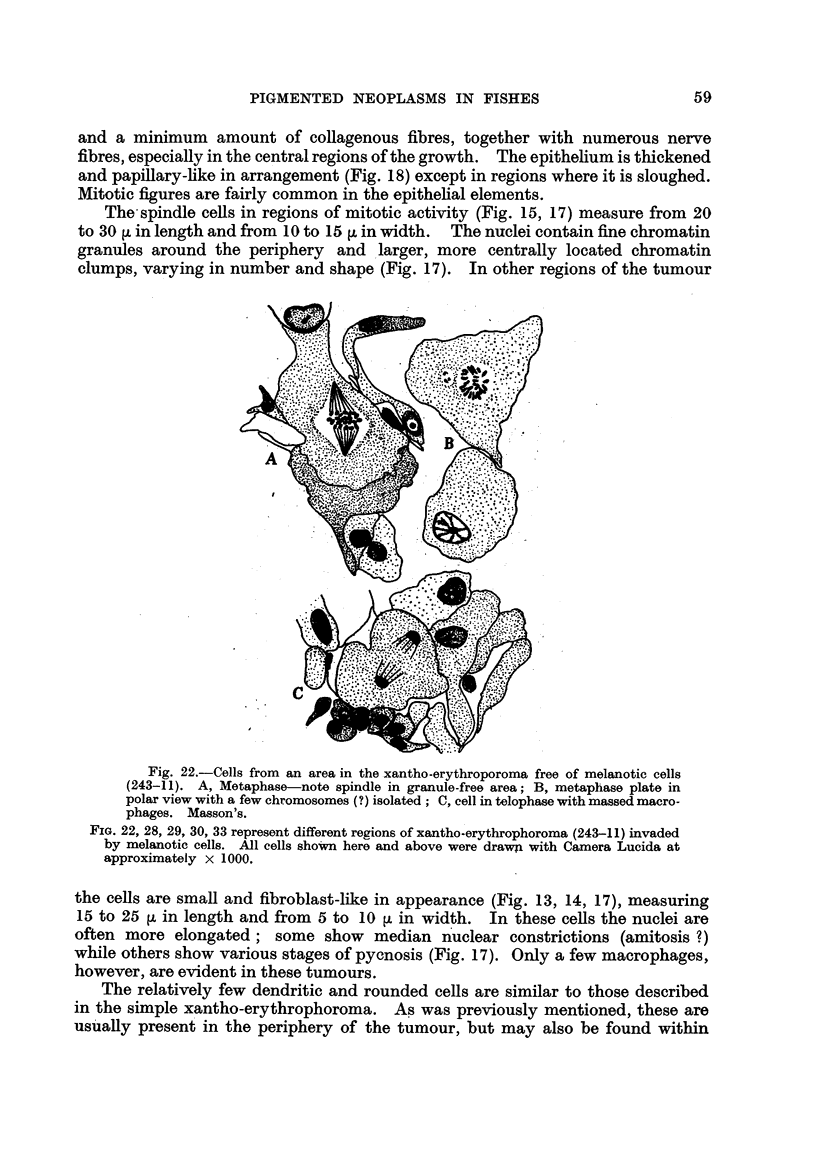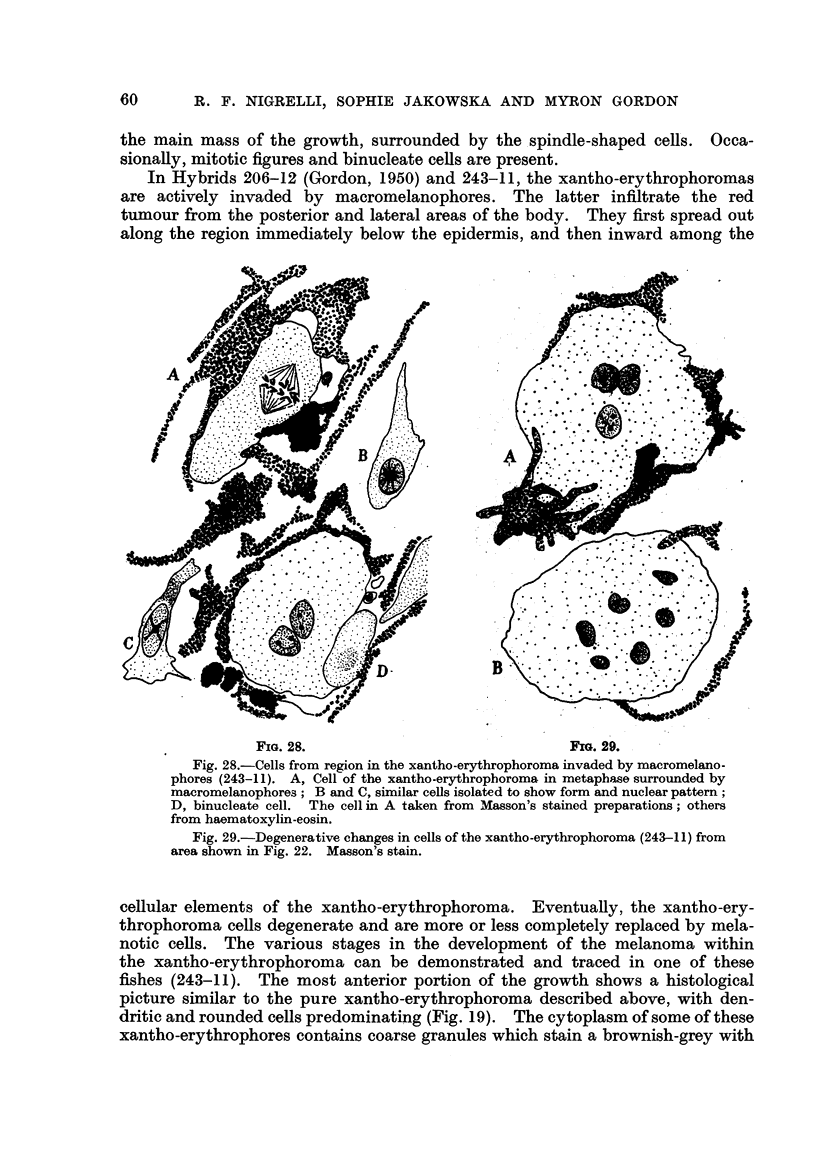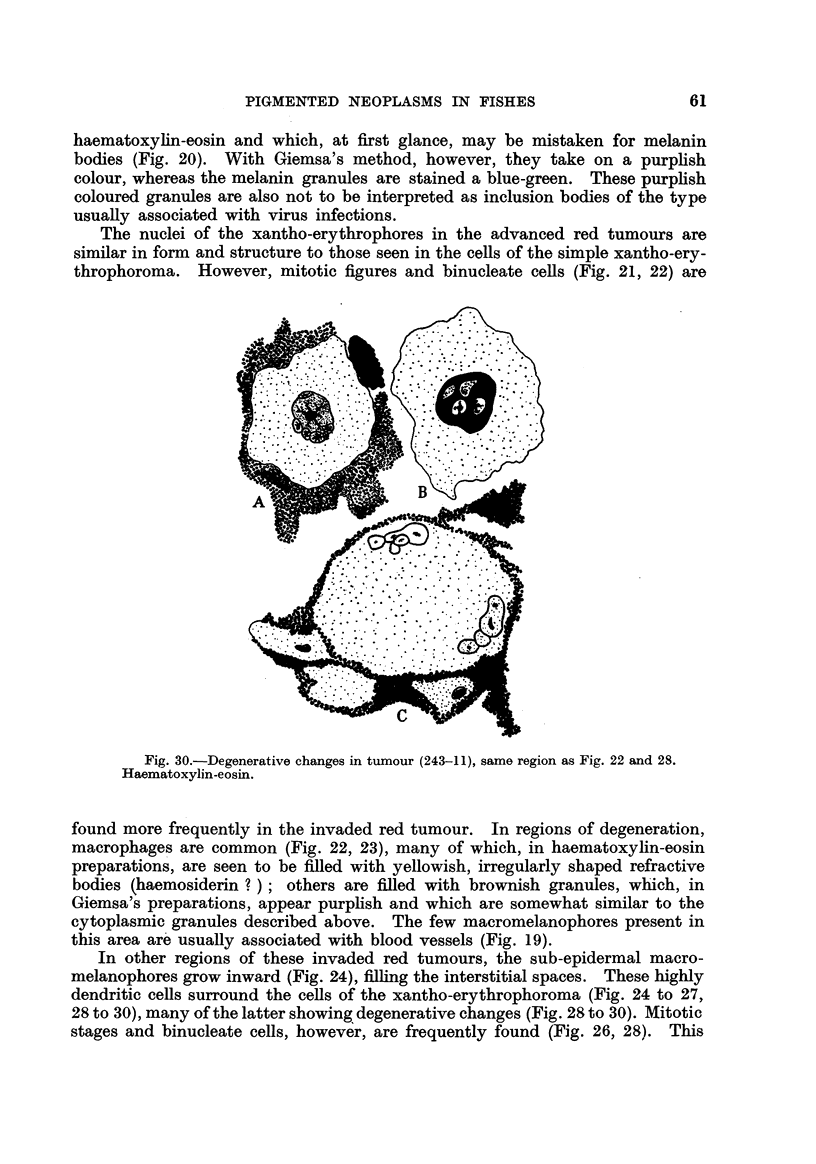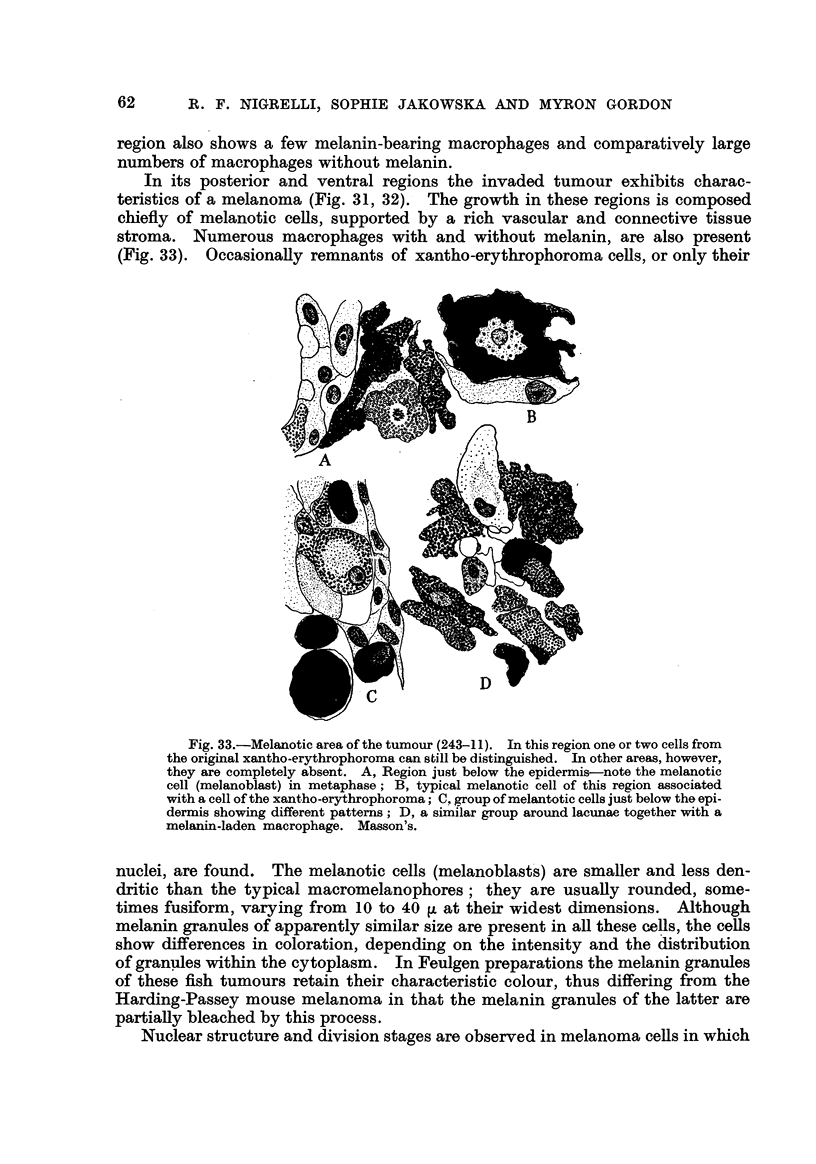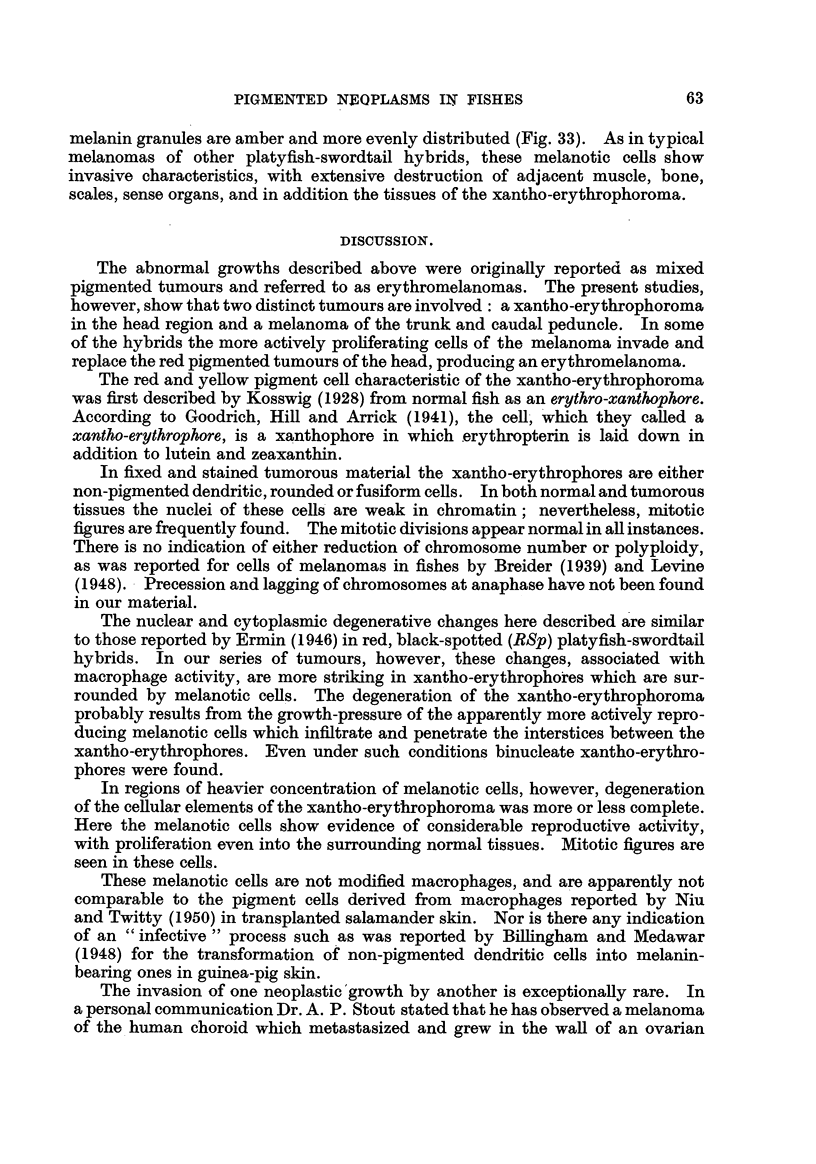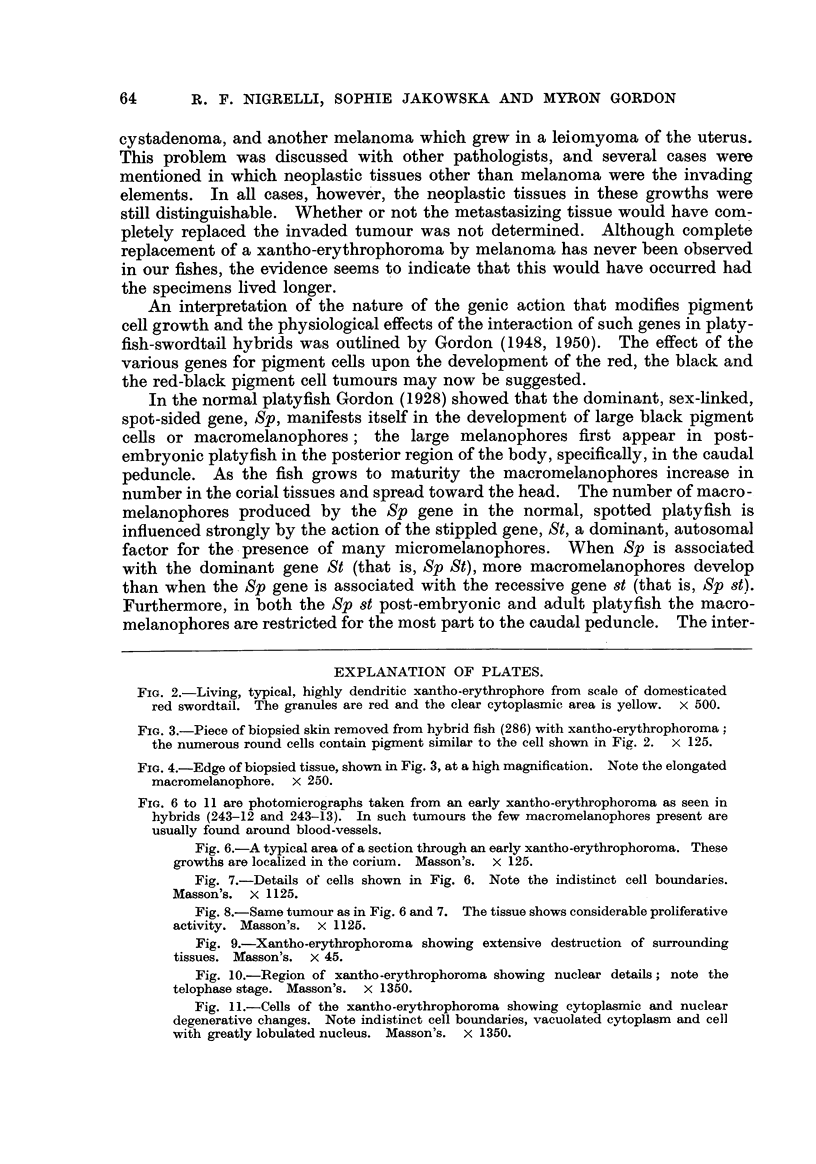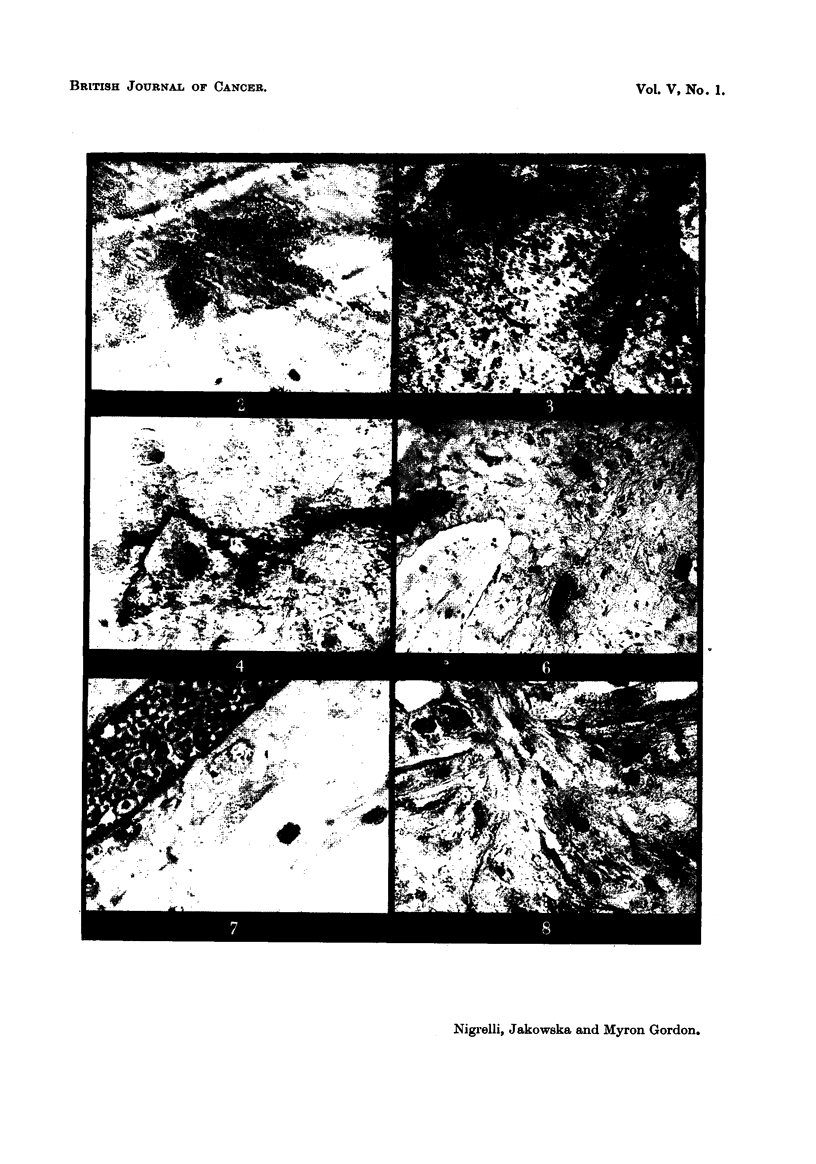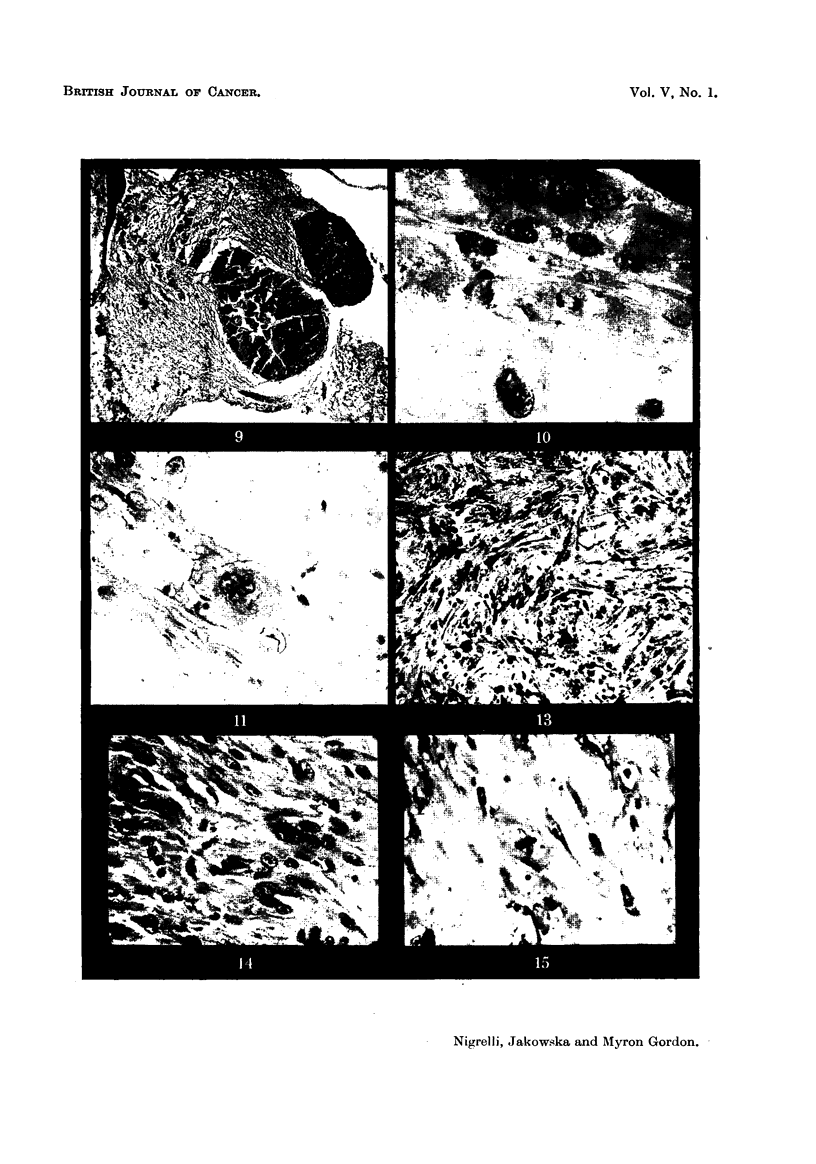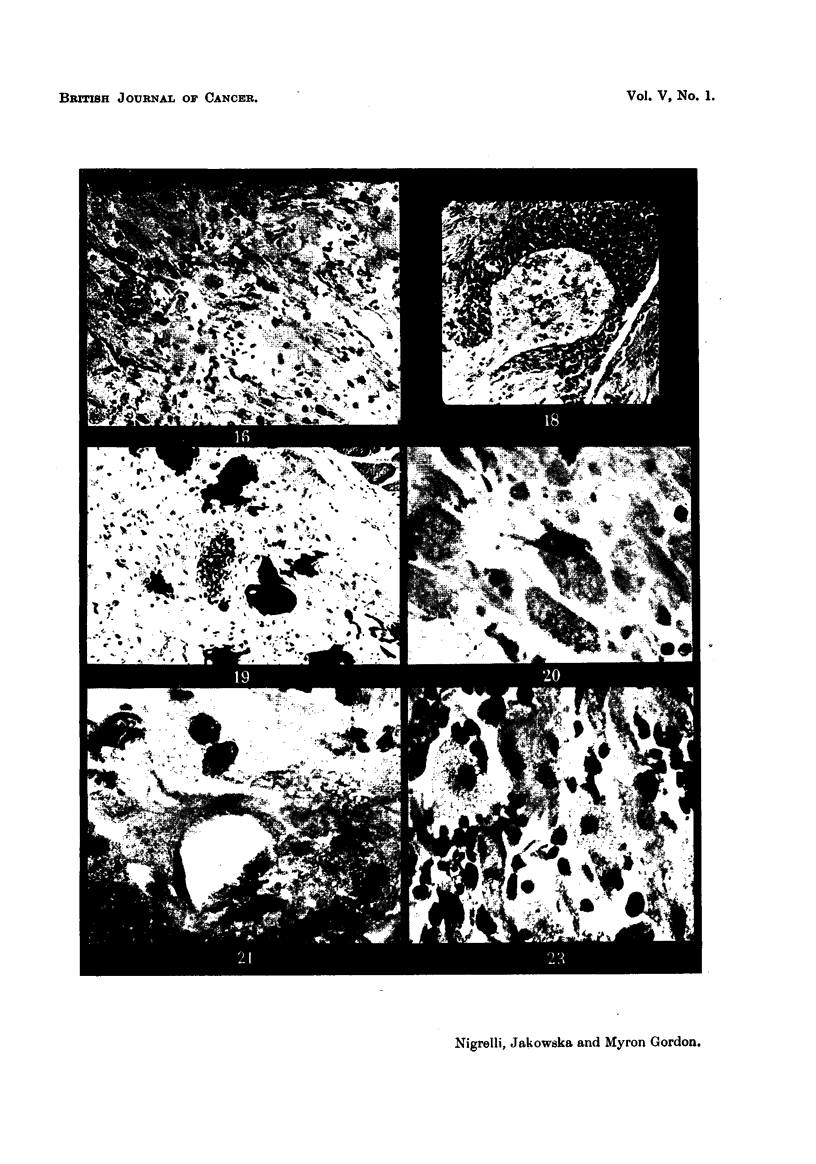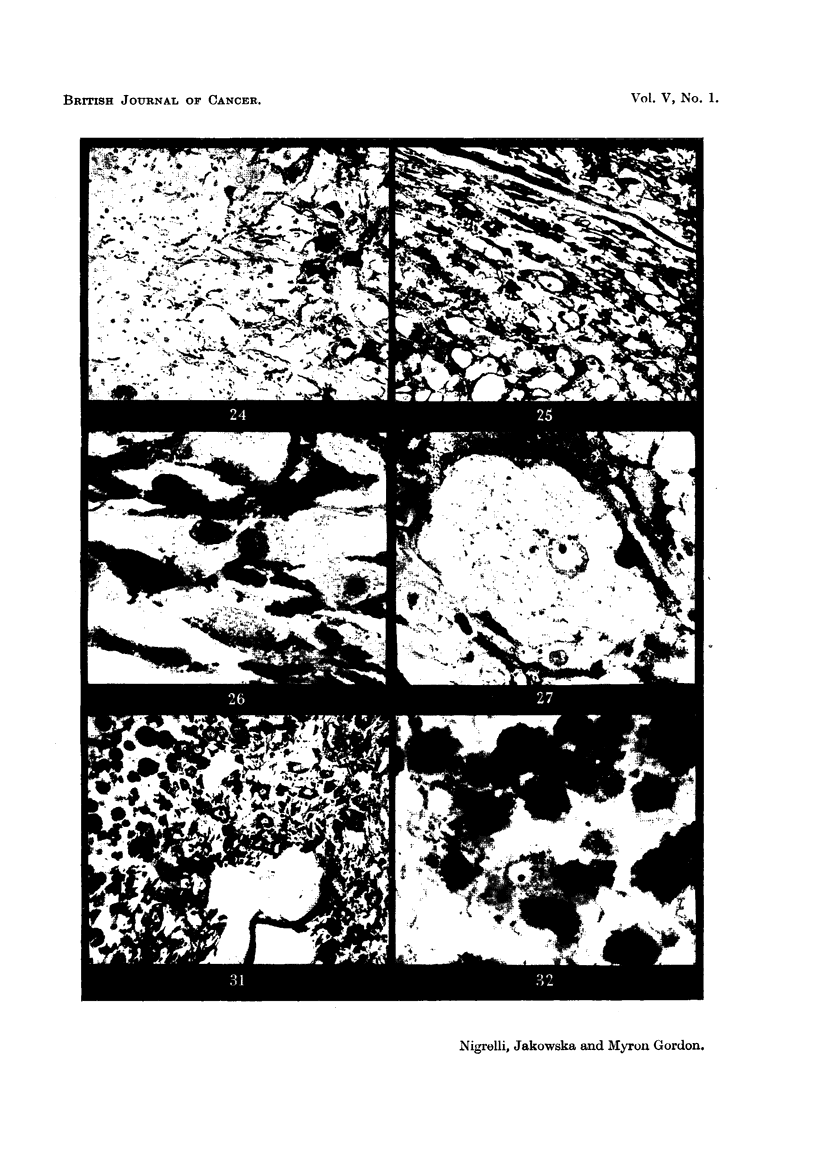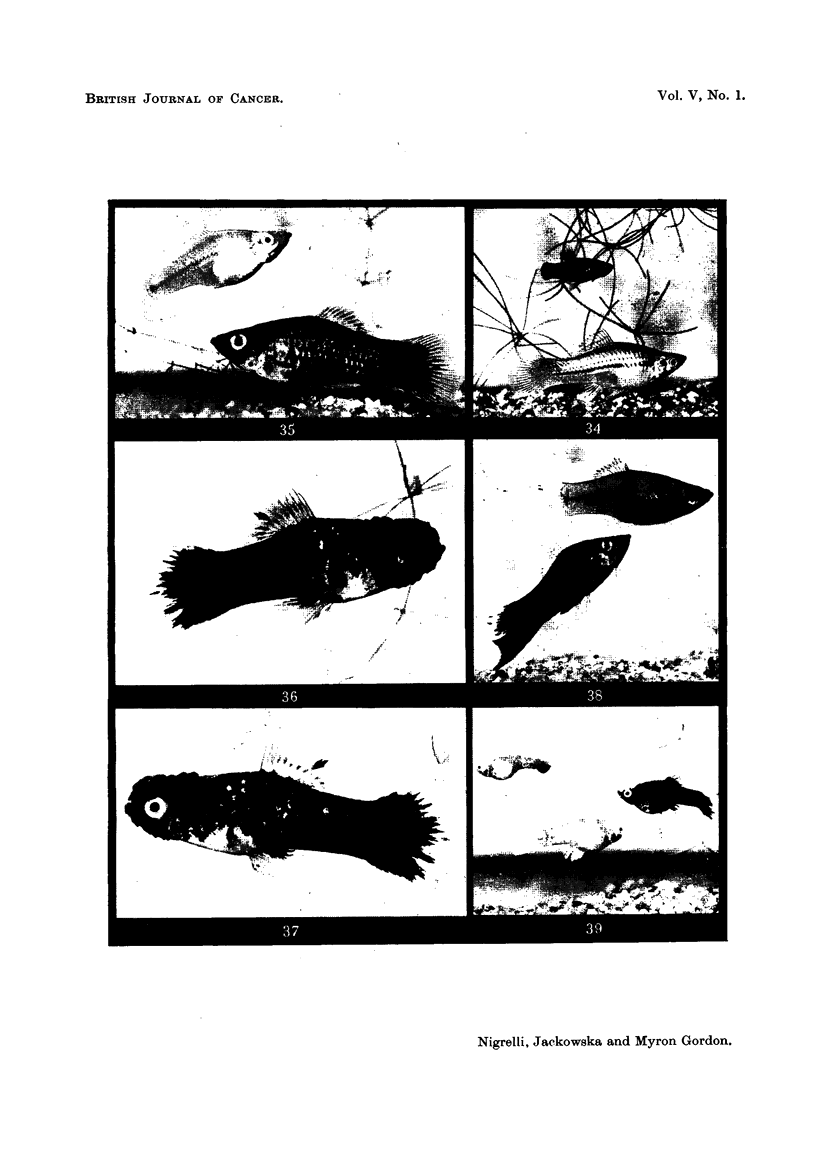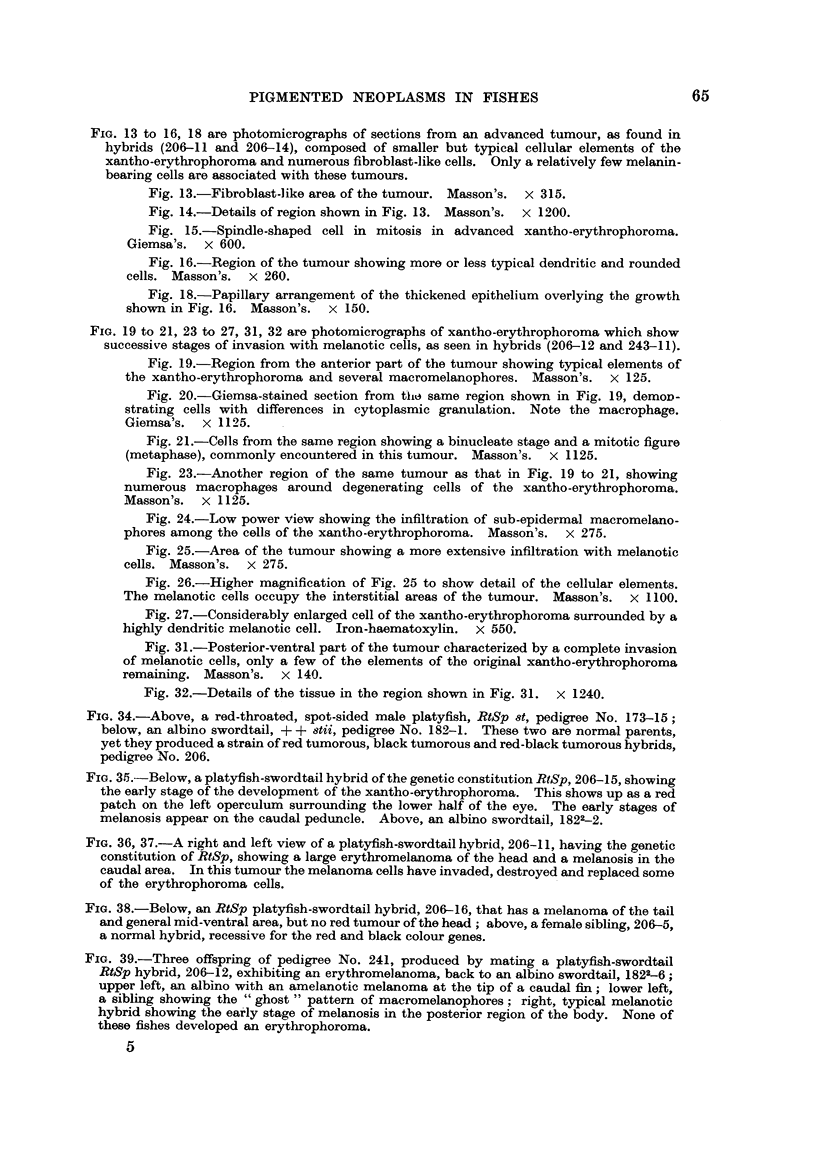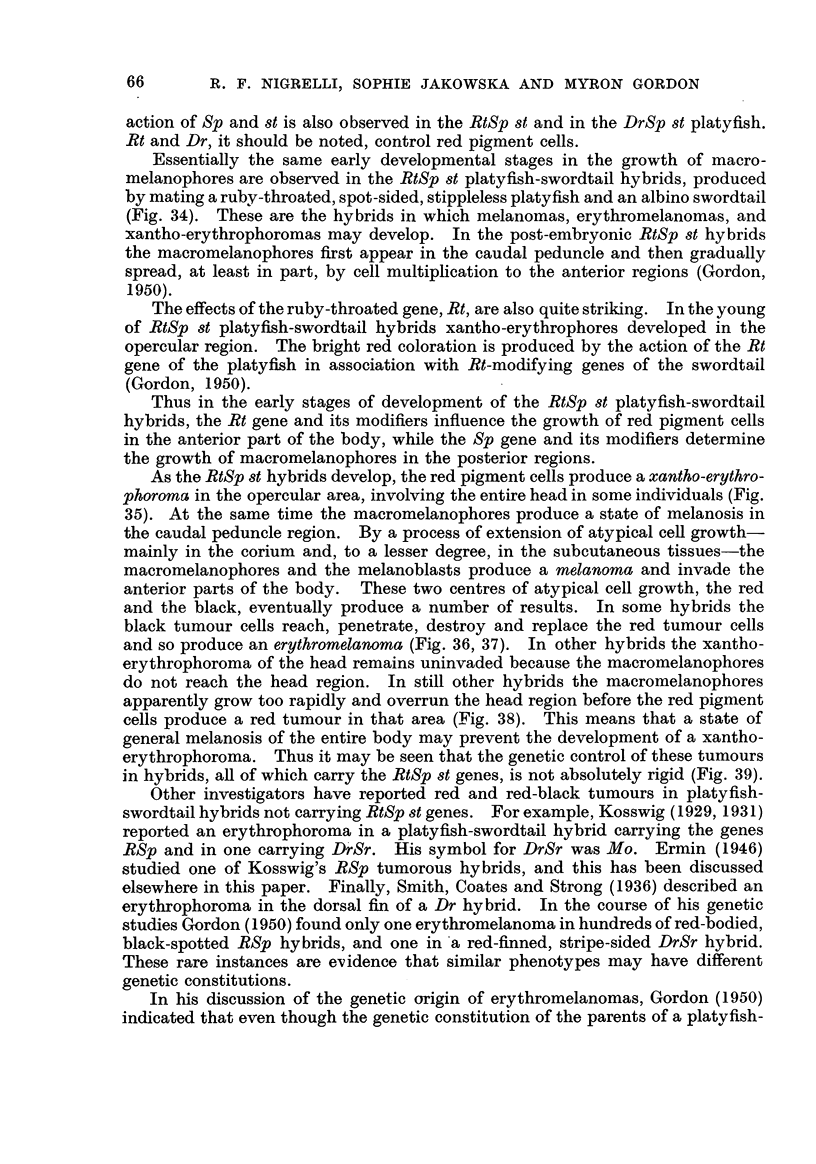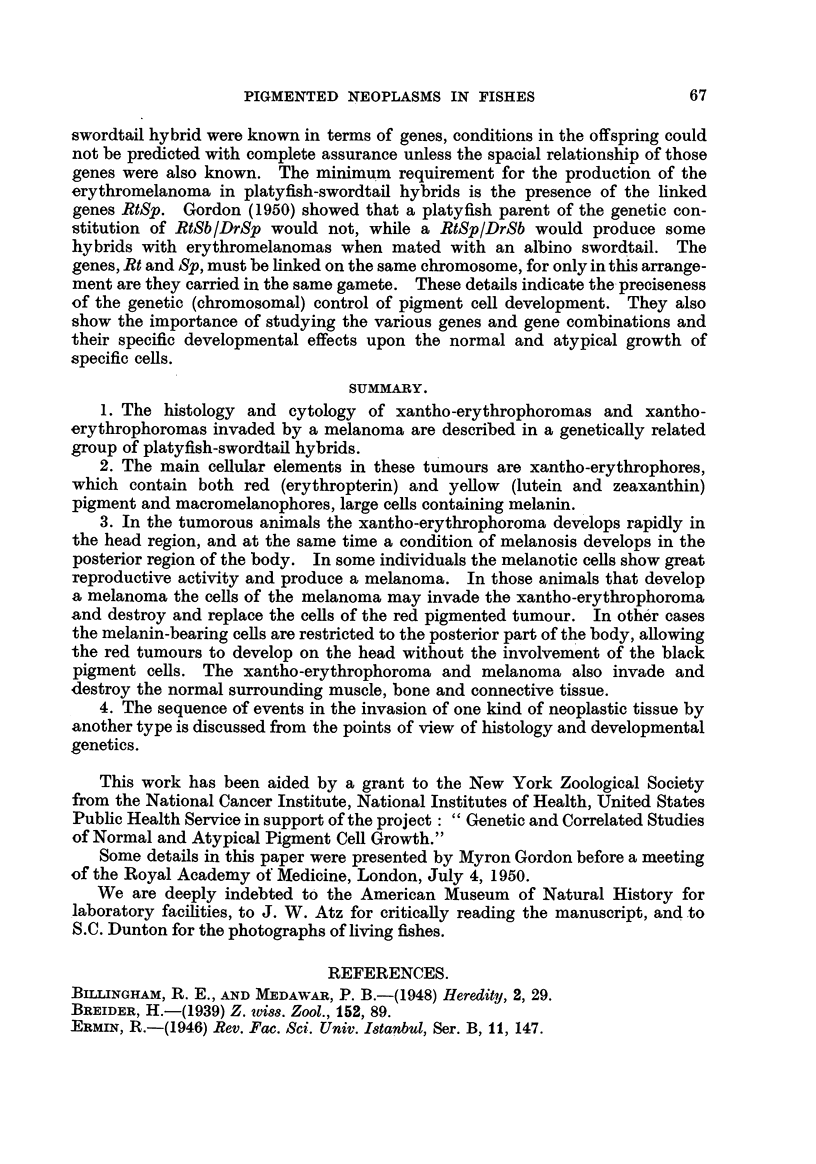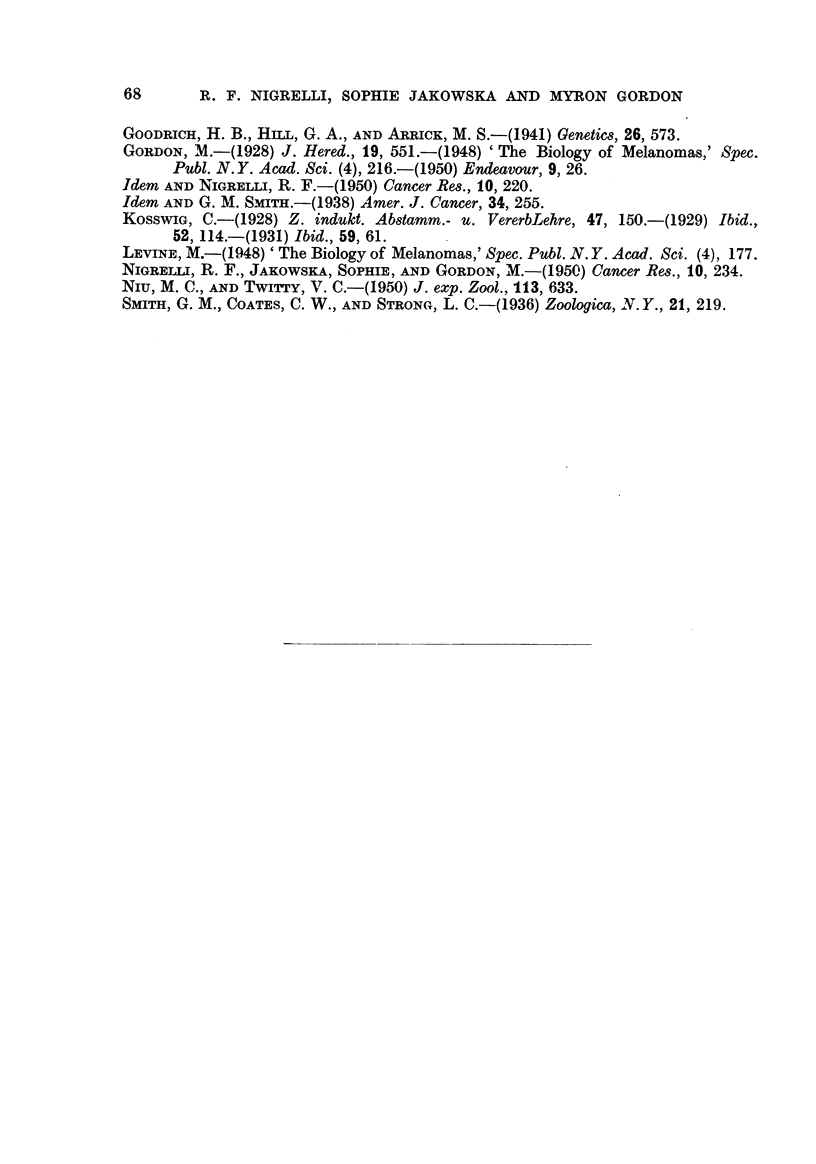# The Invasion and Cell Replacement of One Pigmented Neoplastic Growth by a Second, and More Malignant Type in Experimental Fishes

**DOI:** 10.1038/bjc.1951.6

**Published:** 1951-03

**Authors:** R. F. Nigrelli, Sophie Jakowska, Myron Gordon

## Abstract

**Images:**


### THE INVASION AND CELL REPLACEMENT OF ONE PIGMENTED NEOPLASTIC GROWTH BY A SECOND, AND MORE MALIGNANT TYPE IN EXPERIMENTAL FISHES.

R. F. NIGRELLI, SOPHIE JAKOWSKA AND MYRON GORDON.

*From the New York Aquarium, New York Zoological Society and College of Mount Saint Vincent.*

Received for publication December 21, 1950.

THE invasion and replacement of one kind of tumour by another has rarely been reported in biological studies of neoplastic diseases. Such a phenomenon, involving the abnormal growth of two kinds of pigmented cells, was recently found in spontaneous tumours experimentally produced by genetic methods in platyfish-swordtail hybrids.

The tumorous hybrids were produced by mating a male spot-sided and ruby-throated platyfish (*Platypoecilus maculatus*) to an albino swordtail (*Xiphophorus hellerii*). The spot-sided pattern in the parent platyfish is made up of scattered macromelanophores, which are large black pigment cells containing melanin granules. The ruby-throated pattern is composed of xantho-erythrophores, which are red pigment cells containing erythropterin in granular form and lutein and zeaxanthin in solution. Gordon (1950) and Gordon and Nigrelli (1950) indicated that these colour patterns are inherited and referable to two platyfish sex-linked genes : *Sp* for the black and *Rt* for the red pigment cells. The genetic analysis revealed that the pigmented tumours were produced in platyfish-swordtail hybrids containing the linked genes, *Sp* and *Rt* from the platyfish and a number of *Sp* and *Rt* gene modifiers from the swordtail.

Some of these pigmented tumours were diagnosed as erythromelanomas in an earlier report by Gordon (1950) and Nigrelli, Jakowska and Gordon (1950). More extensive study of additional specimens revealed that there were two kinds of pigmented tumours involved, first, a red tumour, a xantho-erythrophoroma of the head and, second, a black tumour, a melanoma of the body. In some of the hybrids, cells of the melanoma reached, penetrated and replaced the tissues of the red pigmented tumour. When joined, the two pigmented tumours produced an erythromelanoma. The purpose of this paper is to present a more detailed histological and cytological analysis of these pigmented tumours and to suggest a genetic interpretation of the developmental aspects of the problem.

### MATERIAL AND METHODS.

The genetic history of the strain of hybrid fishes that spontaneously developed red tumours was reported by Gordon (1950), a summary of which is indicated in Fig. 1.

Eight platyfish-swordtail hybrids with red or red-black tumours were found in a single brood in the first generation. Of these, 3 are described in this histological analysis. In the second generation 11 hybrids had unmistakable signs of red tumours, of which 3 are here described. In a backcross of one of the tumorous first generation hybrids to a normal swordtail, 5 additional individuals had tumours. When one of the second generation males was bred back to a normal swordtail, 3 more hybrids developed the same kind of pigment cell tumours, one of which is here described.

A total of 27 fishes with red or red-black tumours was obtained by genetic methods. It was not feasible to section all the animals with the red abnormal

FIG. 1.—*First line :* Parental types : 173–15 represents the normal, red-throated, spot-sided male platyfish ; 182–1 represents the normal, albino female swordtail (Fig. 34). *Second line :* First generation platyfish-swordtail hybrids : 206–11 developed a xantho-erythrophoroma ; 206–12 developed an erythromelanoma (Fig. 36, 37) ; 206–1 is a normal recessive hybrid (Fig. 38). *Third line :* Second generation hybrids (pedigree No. 243) to the left and backcross generation hybrids (pedigree No. 241) to the right. (Hybrids of pedigree No. 241 are illustrated by Fig. 39). *Fourth line :* Backcross generation of pedigree No. 286, produced by the mating of a second generation hybrid, 243–14, to an albino swordtail.

□ Male. ○ Female. ◇ Indeterminate. ◆ Tumorous.

growths, but 7 representative specimens were chosen, fixed, sectioned and studied. The first generation hybrids so studied had the following numbers : 206–11, 206–12 and 206–14. The second generation hybrids were 243–11, 243–12 and 243–13. The backcross offspring of a second generation hybrid mated to a swordtail was 286. Some of these fishes are illustrated in Fig. 34 to 39.

As Gordon (1950) indicated, not all hybrids carrying *RtSp* developed typical erythromelanomas or xantho-erythrophoromas. Many of these animals were classified as being in a state of melanosis or simple melanoma. These melanotic animals presented features previously described by Gordon and Smith (1938) and Levine (1948) and are not further described here.

Tumorous fish were first biopsied and later autopsied for microscopical examination. The biopsied material was studied in the fresh state with ordinary light and by phase contrast. In order to obtain better cellular details in the non-fixed material and to identify the pigments, pieces of skin and isolated scales were treated with acetone to extract the xanthophyll and with dilute ammonia to remove the pterins from the pigmented cells. This was the initial procedure recommended by Goodrich, Hill and Arrick (1941) for the extraction and chemical identification of these pigments from the skin of fishes.

FIG. 5.—Normal xantho-erythrophores from isolated scales of a domesticated red swordtail. Haematoxylin eosin. × 1000.

Tissues were fixed in formalin or in Bouin's solution. They were decalcified, embedded in paraffin and sectioned at 5 $\mu$. Sections were stained with Heidenhain's iron-haematoxylin, Harris's haematoxylin and Delafield's haematoxylin, each with and without eosin. Mallory's, Masson's, and Giemsa's staining methods were also used. Material used for Feulgen smears was fixed in 3:1 alcohol-acetic acid solution. Parts of the tumours were treated in bulk for other Feulgen preparations, embedded in paraffin and sectioned.

### HISTOLOGICAL AND CYTOLOGICAL OBSERVATIONS.

The *RtSp* platyfish-swordtail hybrids of the present series show several possible courses of development. Some may develop melanomas, others may develop xantho-erythrophoromas in the opercular area of the head. The red tumours may be invaded by melanotic cells and eventually replaced in part by melanoma cells while others may continue to grow as xantho-erythrophoromas.

The normal xantho-erythrophore in the living, domesticated red swordtail is a highly dendritic cell with coarse red granules and with yellow pigment diffused in the cytoplasm (Fig. 2). When xantho-erythrophores are involved in the tumour formation as, for example, in Hybrid 286, they are pleomorphic ; those that are isolated from the main tumour mass round up and disintegrate rapidly (Fig. 3, 4). In fixed and stained isolated scales from normal skin, the xantho-erythrophores show no dendrites. Their nuclei are spherical or oval ; they stain weakly and contain fine chromatin granules with two or more darker staining bodies (Fig. 5).

Histologically, the simple xantho-erythrophoromas, as represented in Hybrids 243–12 and 243–13 (Gordon, 1950), occupy the corial region of the skin of the

FIG. 12.—Cells from early stage of xantho-erythrophoroma (243–13). A–D, Cells showing different nuclear patterns ; E, cells showing binucleated condition and pycnosis. Note macrophage. Masson's. × 1000.

head (Fig. 6). The pigmented growths are well vascularized (Fig. 6, 7) and the epithelium is often thin or sloughed, probably as the result of pressure from the growing tumour. There is some proliferation into adjacent subcutaneous areas, the growing tissue breaking through limiting membranes (Fig. 8) and causing extensive destruction of muscle and bone (Fig. 9).

The cellular elements of the xantho-erythrophoroma are dendritic, rounded or sometimes fusiform in shape. These cells, whatever their shape, are free of pigment as a result of the techniques employed. They are supported by a delicate vascular and connective tissue stroma (Fig. 6, 7, 8, 10, 11). An occasional macromelanophore may be present, particularly around a blood vessel (Fig. 6). No inflammatory reaction is apparent.

The dendritic, rounded and fusiform cells vary in size from 15 to 90 μ at their largest dimensions (Fig. 12). The cytoplasm is finely or coarsely granular (Fig.

7, 8, 10). The nuclei react weakly to staining and may show one or more larger chromatin bodies (Fig. 7, 8, 10, 12). This weak reaction of the nuclei is also noted in Feulgen treated material. Mitotic figures (Fig. 10) as well as degenerative changes are fairly common in these cells. These changes are manifested by vacuolization of the cytoplasm and by the presence of vacuolated, pycnotic, lobulated and fragmented nuclei (Fig. 11). There seems to be no direct relation between the condition of the cytoplasm and that of the nuclei. Cells with either normal nuclei and vacuolated cytoplasm, or with finely granular cytoplasm and

FIG. 17.—Cells from advanced xantho-erythrophoroma (206–11). A–C, Fusiform cells—note mitotic figure in C; D, group of spindle-shaped cells from another region of the tumour showing several stages of pycnosis. A, B and D stained with haemotoxylin-eosin; C with Giemsa's. × 1000.

nuclei in extreme stages of degeneration are found with equal frequency. Cell boundaries are often indistinct and the cells in many instances simulate a syncytium, independently of nuclear changes (Fig. 7, 8, 10, 11). Macrophage activity is sometimes evident around degenerating cells.

In more advanced xantho-erythrophoromas, as represented by Hybrids 206–11 and 206–14 (Gordon, 1950) spindle and fusiform-shaped cells predominate (Fig. 13 to 17). The typical dendritic and rounded cells are relatively fewer in this stage than in the former, and they are usually found at the periphery of the growth (Fig. 16, 18). Only a few melanin-bearing cells are found scattered throughout the tissue. These tumours have a well developed vascular reticulum and a minimum amount of collagenous fibres, together with numerous nerve fibres, especially in the central regions of the growth. The epithelium is thickened and papillary-like in arrangement (Fig. 18) except in regions where it is sloughed. Mitotic figures are fairly common in the epithelial elements.

The spindle cells in regions of mitotic activity (Fig. 15, 17) measure from 20 to 30 μ in length and from 10 to 15 μ in width. The nuclei contain fine chromatin granules around the periphery and larger, more centrally located chromatin clumps, varying in number and shape (Fig. 17). In other regions of the tumour

Fig. 22.—Cells from an area in the xantho-erythroporoma free of melanotic cells (243–11). A, Metaphase—note spindle in granule-free area; B, metaphase plate in polar view with a few chromosomes (?) isolated; C, cell in telophase with massed macrophages. Masson's.

Fig. 22, 28, 29, 30, 33 represent different regions of xantho-erythrophoroma (243–11) invaded by melanotic cells. All cells shown here and above were drawn with Camera Lucida at approximately × 1000.

the cells are small and fibroblast-like in appearance (Fig. 13, 14, 17), measuring 15 to 25 μ in length and from 5 to 10 μ in width. In these cells the nuclei are often more elongated; some show median nuclear constrictions (amitosis ?) while others show various stages of pycnosis (Fig. 17). Only a few macrophages, however, are evident in these tumours.

The relatively few dendritic and rounded cells are similar to those described in the simple xantho-erythrophoroma. As was previously mentioned, these are usually present in the periphery of the tumour, but may also be found within the main mass of the growth, surrounded by the spindle-shaped cells. Occasionally, mitotic figures and binucleate cells are present.

In Hybrids 206–12 (Gordon, 1950) and 243–11, the xantho-erythrophoromas are actively invaded by macromelanophores. The latter infiltrate the red tumour from the posterior and lateral areas of the body. They first spread out along the region immediately below the epidermis, and then inward among the

FIG. 28.

FIG. 29.

Fig. 28.—Cells from region in the xantho-erythrophoroma invaded by macromelanophores (243–11). A, Cell of the xantho-erythrophoroma in metaphase surrounded by macromelanophores ; B and C, similar cells isolated to show form and nuclear pattern ; D, binucleate cell. The cell in A taken from Masson's stained preparations ; others from haematoxylin-eosin.

Fig. 29.—Degenerative changes in cells of the xantho-erythrophoroma (243–11) from area shown in Fig. 22. Masson's stain.

cellular elements of the xantho-erythrophoroma. Eventually, the xantho-erythrophoroma cells degenerate and are more or less completely replaced by melanotic cells. The various stages in the development of the melanoma within the xantho-erythrophoroma can be demonstrated and traced in one of these fishes (243–11). The most anterior portion of the growth shows a histological picture similar to the pure xantho-erythrophoroma described above, with dendritic and rounded cells predominating (Fig. 19). The cytoplasm of some of these xantho-erythrophores contains coarse granules which stain a brownish-grey with haematoxylin-eosin and which, at first glance, may be mistaken for melanin bodies (Fig. 20). With Giemsa's method, however, they take on a purplish colour, whereas the melanin granules are stained a blue-green. These purplish coloured granules are also not to be interpreted as inclusion bodies of the type usually associated with virus infections.

The nuclei of the xantho-erythrophores in the advanced red tumours are similar in form and structure to those seen in the cells of the simple xantho-erythrophoroma. However, mitotic figures and binucleate cells (Fig. 21, 22) are

Fig. 30.—Degenerative changes in tumour (243–11), same region as Fig. 22 and 28. Haematoxylin-eosin.

found more frequently in the invaded red tumour. In regions of degeneration, macrophages are common (Fig. 22, 23), many of which, in haematoxylin-eosin preparations, are seen to be filled with yellowish, irregularly shaped refractive bodies (haemosiderin ? ) ; others are filled with brownish granules, which, in Giemsa's preparations, appear purplish and which are somewhat similar to the cytoplasmic granules described above. The few macromelanophores present in this area are usually associated with blood vessels (Fig. 19).

In other regions of these invaded red tumours, the sub-epidermal macromelanophores grow inward (Fig. 24), filling the interstitial spaces. These highly dendritic cells surround the cells of the xantho-erythrophoroma (Fig. 24 to 27, 28 to 30), many of the latter showing degenerative changes (Fig. 28 to 30). Mitotic stages and binucleate cells, however, are frequently found (Fig. 26, 28). This region also shows a few melanin-bearing macrophages and comparatively large numbers of macrophages without melanin.

In its posterior and ventral regions the invaded tumour exhibits characteristics of a melanoma (Fig. 31, 32). The growth in these regions is composed chiefly of melanotic cells, supported by a rich vascular and connective tissue stroma. Numerous macrophages with and without melanin, are also present (Fig. 33). Occasionally remnants of xantho-erythrophoroma cells, or only their

Fig. 33.—Melanotic area of the tumour (243–11). In this region one or two cells from the original xantho-erythrophoroma can still be distinguished. In other areas, however, they are completely absent. A, Region just below the epidermis—note the melanotic cell (melanoblast) in metaphase ; B, typical melanotic cell of this region associated with a cell of the xantho-erythrophoroma ; C, group of melantotic cells just below the epidermis showing different patterns ; D, a similar group around lacunae together with a melanin-laden macrophage. Masson's.

nuclei, are found. The melanotic cells (melanoblasts) are smaller and less dendritic than the typical macromelanophores ; they are usually rounded, sometimes fusiform, varying from 10 to 40 $\mu$ at their widest dimensions. Although melanin granules of apparently similar size are present in all these cells, the cells show differences in coloration, depending on the intensity and the distribution of granules within the cytoplasm. In Feulgen preparations the melanin granules of these fish tumours retain their characteristic colour, thus differing from the Harding-Passey mouse melanoma in that the melanin granules of the latter are partially bleached by this process.

Nuclear structure and division stages are observed in melanoma cells in which melanin granules are amber and more evenly distributed (Fig. 33). As in typical melanomas of other platyfish-swordtail hybrids, these melanotic cells show invasive characteristics, with extensive destruction of adjacent muscle, bone, scales, sense organs, and in addition the tissues of the xantho-erythrophoroma.

### DISCUSSION.

The abnormal growths described above were originally reported as mixed pigmented tumours and referred to as erythromelanomas. The present studies, however, show that two distinct tumours are involved : a xantho-erythrophoroma in the head region and a melanoma of the trunk and caudal peduncle. In some of the hybrids the more actively proliferating cells of the melanoma invade and replace the red pigmented tumours of the head, producing an erythromelanoma.

The red and yellow pigment cell characteristic of the xantho-erythrophoroma was first described by Kosswig (1928) from normal fish as an *erythro-xanthophore*. According to Goodrich, Hill and Arrick (1941), the cell, which they called a *xantho-erythrophore*, is a xanthophore in which erythropterin is laid down in addition to lutein and zeaxanthin.

In fixed and stained tumorous material the xantho-erythrophores are either non-pigmented dendritic, rounded or fusiform cells. In both normal and tumorous tissues the nuclei of these cells are weak in chromatin ; nevertheless, mitotic figures are frequently found. The mitotic divisions appear normal in all instances. There is no indication of either reduction of chromosome number or polyploidy, as was reported for cells of melanomas in fishes by Breider (1939) and Levine (1948). Precession and lagging of chromosomes at anaphase have not been found in our material.

The nuclear and cytoplasmic degenerative changes here described are similar to those reported by Ermin (1946) in red, black-spotted (*RSp*) platyfish-swordtail hybrids. In our series of tumours, however, these changes, associated with macrophage activity, are more striking in xantho-erythrophores which are surrounded by melanotic cells. The degeneration of the xantho-erythrophoroma probably results from the growth-pressure of the apparently more actively reproducing melanotic cells which infiltrate and penetrate the interstices between the xantho-erythrophores. Even under such conditions binucleate xantho-erythrophores were found.

In regions of heavier concentration of melanotic cells, however, degeneration of the cellular elements of the xantho-erythrophoroma was more or less complete. Here the melanotic cells show evidence of considerable reproductive activity, with proliferation even into the surrounding normal tissues. Mitotic figures are seen in these cells.

These melanotic cells are not modified macrophages, and are apparently not comparable to the pigment cells derived from macrophages reported by Niu and Twitty (1950) in transplanted salamander skin. Nor is there any indication of an " infective " process such as was reported by Billingham and Medawar (1948) for the transformation of non-pigmented dendritic cells into melanin-bearing ones in guinea-pig skin.

The invasion of one neoplastic growth by another is exceptionally rare. In a personal communication Dr. A. P. Stout stated that he has observed a melanoma of the human choroid which metastasized and grew in the wall of an ovarian cystadenoma, and another melanoma which grew in a leiomyoma of the uterus. This problem was discussed with other pathologists, and several cases were mentioned in which neoplastic tissues other than melanoma were the invading elements. In all cases, however, the neoplastic tissues in these growths were still distinguishable. Whether or not the metastasizing tissue would have completely replaced the invaded tumour was not determined. Although complete replacement of a xantho-erythrophoroma by melanoma has never been observed in our fishes, the evidence seems to indicate that this would have occurred had the specimens lived longer.

An interpretation of the nature of the genic action that modifies pigment cell growth and the physiological effects of the interaction of such genes in platyfish-swordtail hybrids was outlined by Gordon (1948, 1950). The effect of the various genes for pigment cells upon the development of the red, the black and the red-black pigment cell tumours may now be suggested.

In the normal platyfish Gordon (1928) showed that the dominant, sex-linked, spot-sided gene, *Sp*, manifests itself in the development of large black pigment cells or macromelanophores; the large melanophores first appear in post-embryonic platyfish in the posterior region of the body, specifically, in the caudal peduncle. As the fish grows to maturity the macromelanophores increase in number in the corial tissues and spread toward the head. The number of macromelanophores produced by the *Sp* gene in the normal, spotted platyfish is influenced strongly by the action of the stippled gene, *St*, a dominant, autosomal factor for the presence of many micromelanophores. When *Sp* is associated with the dominant gene *St* (that is, *Sp St*), more macromelanophores develop than when the *Sp* gene is associated with the recessive gene *st* (that is, *Sp st*). Furthermore, in both the *Sp st* post-embryonic and adult platyfish the macromelanophores are restricted for the most part to the caudal peduncle. The inter-

---

EXPLANATION OF PLATES.

Fig. 2.—Living, typical, highly dendritic xantho-erythrophore from scale of domesticated red swordtail. The granules are red and the clear cytoplasmic area is yellow. × 500.

Fig. 3.—Piece of biopsied skin removed from hybrid fish (286) with xantho-erythrophoroma; the numerous round cells contain pigment similar to the cell shown in Fig. 2. × 125.

Fig. 4.—Edge of biopsied tissue, shown in Fig. 3, at a high magnification. Note the elongated macromelanophore. × 250.

Fig. 6 to 11 are photomicrographs taken from an early xantho-erythrophoroma as seen in hybrids (243–12 and 243–13). In such tumours the few macromelanophores present are usually found around blood-vessels.

Fig. 6.—A typical area of a section through an early xantho-erythrophoroma. These growths are localized in the corium. Masson's. × 125.

Fig. 7.—Details of cells shown in Fig. 6. Note the indistinct cell boundaries. Masson's. × 1125.

Fig. 8.—Same tumour as in Fig. 6 and 7. The tissue shows considerable proliferative activity. Masson's. × 1125.

Fig. 9.—Xantho-erythrophoroma showing extensive destruction of surrounding tissues. Masson's. × 45.

Fig. 10.—Region of xantho-erythrophoroma showing nuclear details; note the telophase stage. Masson's. × 1350.

Fig. 11.—Cells of the xantho-erythrophoroma showing cytoplasmic and nuclear degenerative changes. Note indistinct cell boundaries, vacuolated cytoplasm and cell with greatly lobulated nucleus. Masson's. × 1350.

Nigrelli, Jakowska and Myron Gordon.

9

10

11

13

14

15

Nigrelli, Jakowska and Myron Gordon.

16

18

19

20

21

23

Nigrelli, Jakowska and Myron Gordon.

24

25

26

27

31

32

Nigrelli, Jakowska and Myron Gordon.

Nigrelli, Jackowska and Myron Gordon.

FIG. 13 to 16, 18 are photomicrographs of sections from an advanced tumour, as found in hybrids (206–11 and 206–14), composed of smaller but typical cellular elements of the xantho-erythrophoroma and numerous fibroblast-like cells. Only a relatively few melanin-bearing cells are associated with these tumours.

Fig. 13.—Fibroblast-like area of the tumour. Masson's. × 315.

Fig. 14.—Details of region shown in Fig. 13. Masson's. × 1200.

Fig. 15.—Spindle-shaped cell in mitosis in advanced xantho-erythrophoroma. Giemsa's. × 600.

Fig. 16.—Region of the tumour showing more or less typical dendritic and rounded cells. Masson's. × 260.

Fig. 18.—Papillary arrangement of the thickened epithelium overlying the growth shown in Fig. 16. Masson's. × 150.

FIG. 19 to 21, 23 to 27, 31, 32 are photomicrographs of xantho-erythrophoroma which show successive stages of invasion with melanotic cells, as seen in hybrids (206–12 and 243–11).

Fig. 19.—Region from the anterior part of the tumour showing typical elements of the xantho-erythrophoroma and several macromelanophores. Masson's. × 125.

Fig. 20.—Giemsa-stained section from the same region shown in Fig. 19, demonstrating cells with differences in cytoplasmic granulation. Note the macrophage. Giemsa's. × 1125.

Fig. 21.—Cells from the same region showing a binucleate stage and a mitotic figure (metaphase), commonly encountered in this tumour. Masson's. × 1125.

Fig. 23.—Another region of the same tumour as that in Fig. 19 to 21, showing numerous macrophages around degenerating cells of the xantho-erythrophoroma. Masson's. × 1125.

Fig. 24.—Low power view showing the infiltration of sub-epidermal macromelanophores among the cells of the xantho-erythrophoroma. Masson's. × 275.

Fig. 25.—Area of the tumour showing a more extensive infiltration with melanotic cells. Masson's. × 275.

Fig. 26.—Higher magnification of Fig. 25 to show detail of the cellular elements. The melanotic cells occupy the interstitial areas of the tumour. Masson's. × 1100.

Fig. 27.—Considerably enlarged cell of the xantho-erythrophoroma surrounded by a highly dendritic melanotic cell. Iron-haematoxylin. × 550.

Fig. 31.—Posterior-ventral part of the tumour characterized by a complete invasion of melanotic cells, only a few of the elements of the original xantho-erythrophoroma remaining. Masson's. × 140.

Fig. 32.—Details of the tissue in the region shown in Fig. 31. × 1240.

FIG. 34.—Above, a red-throated, spot-sided male platyfish, *RtSp st*, pedigree No. 173–15; below, an albino swordtail, + + *stii*, pedigree No. 182–1. These two are normal parents, yet they produced a strain of red tumorous, black tumorous and red-black tumorous hybrids, pedigree No. 206.

FIG. 35.—Below, a platyfish-swordtail hybrid of the genetic constitution *RtSp*, 206–15, showing the early stage of the development of the xantho-erythrophoroma. This shows up as a red patch on the left operculum surrounding the lower half of the eye. The early stages of melanosis appear on the caudal peduncle. Above, an albino swordtail, $182^2$–2.

FIG. 36, 37.—A right and left view of a platyfish-swordtail hybrid, 206–11, having the genetic constitution of *RtSp*, showing a large erythromelanoma of the head and a melanosis in the caudal area. In this tumour the melanoma cells have invaded, destroyed and replaced some of the erythrophoroma cells.

FIG. 38.—Below, an *RtSp* platyfish-swordtail hybrid, 206–16, that has a melanoma of the tail and general mid-ventral area, but no red tumour of the head; above, a female sibling, 206–5, a normal hybrid, recessive for the red and black colour genes.

FIG. 39.—Three offspring of pedigree No. 241, produced by mating a platyfish-swordtail *RtSp* hybrid, 206–12, exhibiting an erythromelanoma, back to an albino swordtail, $182^2$–6; upper left, an albino with an amelanotic melanoma at the tip of a caudal fin; lower left, a sibling showing the " ghost " pattern of macromelanophores; right, typical melanotic hybrid showing the early stage of melanosis in the posterior region of the body. None of these fishes developed an erythrophoroma.

action of *Sp* and *st* is also observed in the *RtSp st* and in the *DrSp st* platyfish. *Rt* and *Dr*, it should be noted, control red pigment cells.

Essentially the same early developmental stages in the growth of macromelanophores are observed in the *RtSp st* platyfish-swordtail hybrids, produced by mating a ruby-throated, spot-sided, stippleless platyfish and an albino swordtail (Fig. 34). These are the hybrids in which melanomas, erythromelanomas, and xantho-erythrophoromas may develop. In the post-embryonic *RtSp st* hybrids the macromelanophores first appear in the caudal peduncle and then gradually spread, at least in part, by cell multiplication to the anterior regions (Gordon, 1950).

The effects of the ruby-throated gene, *Rt*, are also quite striking. In the young of *RtSp st* platyfish-swordtail hybrids xantho-erythrophores developed in the opercular region. The bright red coloration is produced by the action of the *Rt* gene of the platyfish in association with *Rt*-modifying genes of the swordtail (Gordon, 1950).

Thus in the early stages of development of the *RtSp st* platyfish-swordtail hybrids, the *Rt* gene and its modifiers influence the growth of red pigment cells in the anterior part of the body, while the *Sp* gene and its modifiers determine the growth of macromelanophores in the posterior regions.

As the *RtSp st* hybrids develop, the red pigment cells produce a *xantho-erythrophoroma* in the opercular area, involving the entire head in some individuals (Fig. 35). At the same time the macromelanophores produce a state of melanosis in the caudal peduncle region. By a process of extension of atypical cell growth—mainly in the corium and, to a lesser degree, in the subcutaneous tissues—the macromelanophores and the melanoblasts produce a *melanoma* and invade the anterior parts of the body. These two centres of atypical cell growth, the red and the black, eventually produce a number of results. In some hybrids the black tumour cells reach, penetrate, destroy and replace the red tumour cells and so produce an *erythromelanoma* (Fig. 36, 37). In other hybrids the xantho-erythrophoroma of the head remains uninvaded because the macromelanophores do not reach the head region. In still other hybrids the macromelanophores apparently grow too rapidly and overrun the head region before the red pigment cells produce a red tumour in that area (Fig. 38). This means that a state of general melanosis of the entire body may prevent the development of a xantho-erythrophoroma. Thus it may be seen that the genetic control of these tumours in hybrids, all of which carry the *RtSp st* genes, is not absolutely rigid (Fig. 39).

Other investigators have reported red and red-black tumours in platyfish-swordtail hybrids not carrying *RtSp st* genes. For example, Kosswig (1929, 1931) reported an erythrophoroma in a platyfish-swordtail hybrid carrying the genes *RSp* and in one carrying *DrSr*. His symbol for *DrSr* was *Mo*. Ermin (1946) studied one of Kosswig's *RSp* tumorous hybrids, and this has been discussed elsewhere in this paper. Finally, Smith, Coates and Strong (1936) described an erythrophoroma in the dorsal fin of a *Dr* hybrid. In the course of his genetic studies Gordon (1950) found only one erythromelanoma in hundreds of red-bodied, black-spotted *RSp* hybrids, and one in a red-finned, stripe-sided *DrSr* hybrid. These rare instances are evidence that similar phenotypes may have different genetic constitutions.

In his discussion of the genetic origin of erythromelanomas, Gordon (1950) indicated that even though the genetic constitution of the parents of a platyfishswordtail hybrid were known in terms of genes, conditions in the offspring could not be predicted with complete assurance unless the spacial relationship of those genes were also known. The minimum requirement for the production of the erythromelanoma in platyfish-swordtail hybrids is the presence of the linked genes *RtSp*. Gordon (1950) showed that a platyfish parent of the genetic constitution of *RtSb/DrSp* would not, while a *RtSp/DrSb* would produce some hybrids with erythromelanomas when mated with an albino swordtail. The genes, *Rt* and *Sp*, must be linked on the same chromosome, for only in this arrangement are they carried in the same gamete. These details indicate the preciseness of the genetic (chromosomal) control of pigment cell development. They also show the importance of studying the various genes and gene combinations and their specific developmental effects upon the normal and atypical growth of specific cells.

SUMMARY.

1. The histology and cytology of xantho-erythrophoromas and xantho-erythrophoromas invaded by a melanoma are described in a genetically related group of platyfish-swordtail hybrids.

2. The main cellular elements in these tumours are xantho-erythrophores, which contain both red (erythropterin) and yellow (lutein and zeaxanthin) pigment and macromelanophores, large cells containing melanin.

3. In the tumorous animals the xantho-erythrophoroma develops rapidly in the head region, and at the same time a condition of melanosis develops in the posterior region of the body. In some individuals the melanotic cells show great reproductive activity and produce a melanoma. In those animals that develop a melanoma the cells of the melanoma may invade the xantho-erythrophoroma and destroy and replace the cells of the red pigmented tumour. In other cases the melanin-bearing cells are restricted to the posterior part of the body, allowing the red tumours to develop on the head without the involvement of the black pigment cells. The xantho-erythrophoroma and melanoma also invade and destroy the normal surrounding muscle, bone and connective tissue.

4. The sequence of events in the invasion of one kind of neoplastic tissue by another type is discussed from the points of view of histology and developmental genetics.

This work has been aided by a grant to the New York Zoological Society from the National Cancer Institute, National Institutes of Health, United States Public Health Service in support of the project : " Genetic and Correlated Studies of Normal and Atypical Pigment Cell Growth."

Some details in this paper were presented by Myron Gordon before a meeting of the Royal Academy of Medicine, London, July 4, 1950.

We are deeply indebted to the American Museum of Natural History for laboratory facilities, to J. W. Atz for critically reading the manuscript, and to S.C. Dunton for the photographs of living fishes.

REFERENCES.

Billingham, R. E., and Medawar, P. B.—(1948) *Heredity*, **2**, 29.

Breider, H.—(1939) *Z. wiss. Zool.*, **152**, 89.

Ermin, R.—(1946) *Rev. Fac. Sci. Univ. Istanbul*, Ser. B, **11**, 147.

GOODRICH, H. B., HILL, G. A., AND ARRICK, M. S.—(1941) *Genetics*, **26**, 573.
GORDON, M.—(1928) *J. Hered.*, **19**, 551.—(1948) 'The Biology of Melanomas,' *Spec. Publ. N.Y. Acad. Sci.* (4), 216.—(1950) *Endeavour*, **9**, 26.
*Idem* AND NIGRELLI, R. F.—(1950) *Cancer Res.*, **10**, 220.
*Idem* AND G. M. SMITH.—(1938) *Amer. J. Cancer*, **34**, 255.
KOSSWIG, C.—(1928) *Z. indukt. Abstamm.- u. VererbLehre*, **47**, 150.—(1929) *Ibid.*, **52**, 114.—(1931) *Ibid.*, **59**, 61.
LEVINE, M.—(1948) 'The Biology of Melanomas,' *Spec. Publ. N.Y. Acad. Sci.* (4), 177.
NIGRELLI, R. F., JAKOWSKA, SOPHIE, AND GORDON, M.—(1950) *Cancer Res.*, **10**, 234.
NIU, M. C., AND TWITTY, V. C.—(1950) *J. exp. Zool.*, **113**, 633.
SMITH, G. M., COATES, C. W., AND STRONG, L. C.—(1936) *Zoologica, N.Y.*, **21**, 219.